# Supplementary material for: Designing transparent piezoelectric metasurfaces for adaptive optics
Source: Nat Commun. 2024 Jan 27;15:805. doi: 10.1038/s41467-024-45088-3 (PMC10821918; doi:10.1038/s41467-024-45088-3)
Supplement: Supplementary file 1 — Supplementary Information [file 41467_2024_45088_MOESM1_ESM.pdf]

**Supplementary material**

# Designing transparent piezoelectric metasurfaces for adaptive optics

Liao Qiao[1], Xiangyu Gao[1*], Kaile Ren[1], Chaorui Qiu[1], Jinfeng Liu[1], Haonan Jin[1], Shuxiang Dong[2], Zhuo Xu[1*], and Fei Li[1*]

[1]Electronic Materials Research Laboratory, Key Lab of Education Ministry and State Key Laboratory for Mechanical Behavior of Materials, School of Electronic Science and Engineering, Xi'an Jiaotong University, Xi'an, 710049, China.

[2]Institute for Advanced Study, Shenzhen University, Shenzhen, 518051, China.

[*]Corresponding authors. Email: gaoxiangyu@xjtu.edu.cn (X. G.); xuzhuo@xjtu.edu.cn (Z. X.); ful5@xjtu.edu.cn (F. L.)

## Supplementary Note 1. Designing of the piezo metasurfaces (PM) with piezoelectric units.

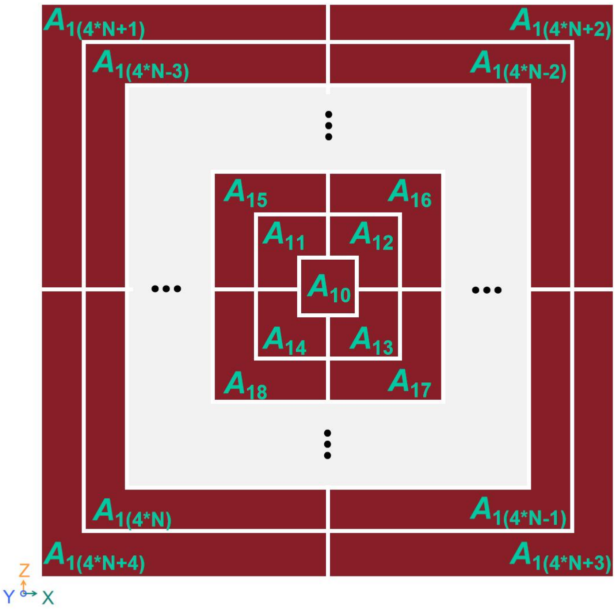


**Supplementary Fig. 1. Schematic diagram of the center outward expansion method.**

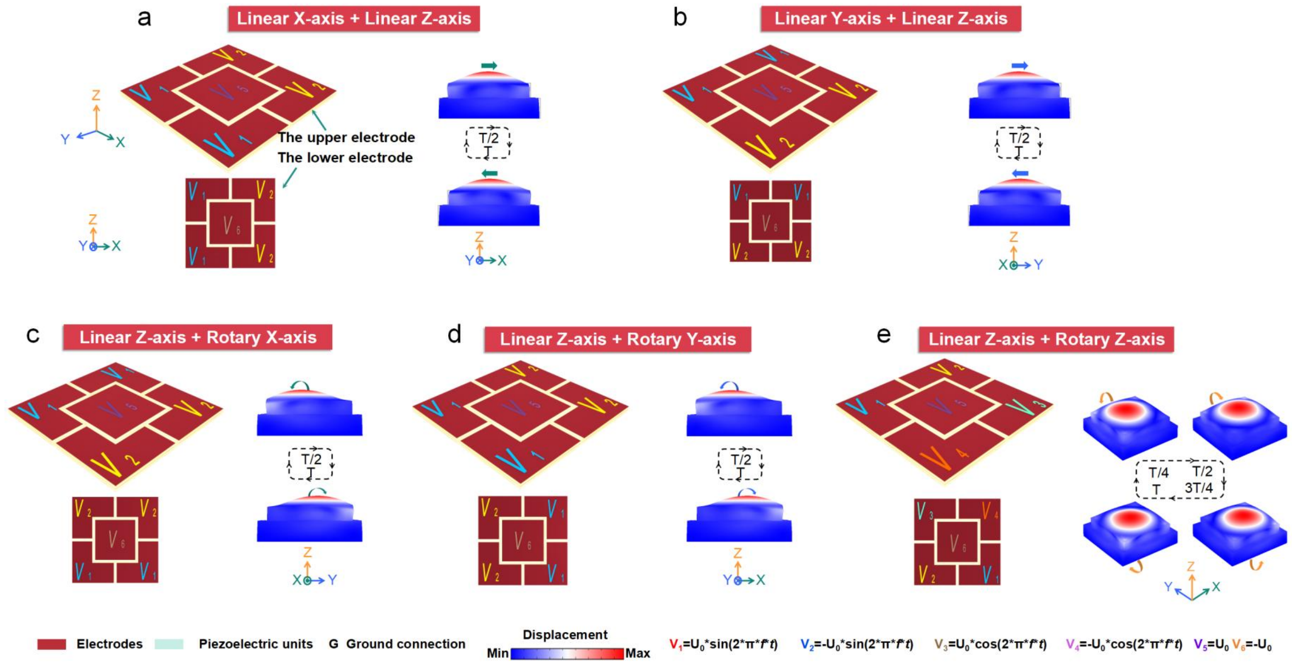


**Supplementary Fig. 2. Design of an (5 × 2) arrayed PM to simultaneously accomplish two motion modes.** (a-e) The applying voltage signals and simulated deformations of simultaneously accomplish (a) the linear motion along X-axis and the linear motion along Z-axis, (b) the linear motion along Y-axis and the linear motion along Z-axis, (c) the linear motion along Z-axis and the rotary motion around X-axis, (d) the linear motion along Z-axis and the rotary motion around Y-axis, (e) the linear motion along Z-axis and the rotary motion around Z-axis in the PM.

Our designed arrayed PM has the ability to simultaneously accomplish multiple motion modes. The detailed method is clarified as follows.

(1) The method for simultaneously accomplishing two motion modes

As shown in **Supplementary Fig. 2**, the piezoelectric units are divided into two groups, where $A_{10}$ and $A_{20}$ are classified as group I, and the $A_{11}$, $A_{12}$, $A_{13}$, $A_{14}$, $A_{21}$, $A_{22}$, $A_{23}$ and $A_{24}$ are classified as

group II. By applying predetermined electric fields, group I realizes the linear motion along the Z-axis, and group II can realize the linear motions along the X- and Y-axis, and/or the rotary motions around the X-, Y- and Z-axis. The two motion modes can be independently excited and thus two motion modes can be simultaneously accomplished.

For example, for simultaneously accomplishing the linear motion along the Z-axis and the linear motion along the X-axis (**Supplementary Fig. 2a**), the $A_{20}$ with voltage signal $V_5$ and the $A_{10}$ with voltage signal $V_6$ are classified as group I, while the $A_{11}$, $A_{14}$, $A_{21}$ and $A_{24}$ units with voltage signal $V_1$ and the $A_{12}$, $A_{13}$, $A_{22}$ and $A_{23}$ units with voltage signal $V_2$ are classified as group II. In this case, various motion modes can be produced for group I and II, i.e., group I generates the arching (or incurvaing) deformation along the positive (or negative) direction of the Z-axis, group II causes the whole PM by the lateral translation deformation along the X-axis. Therefore, simultaneously accomplishing the linear motion along the Z-axis and the linear motion along the X-axis can be realized.

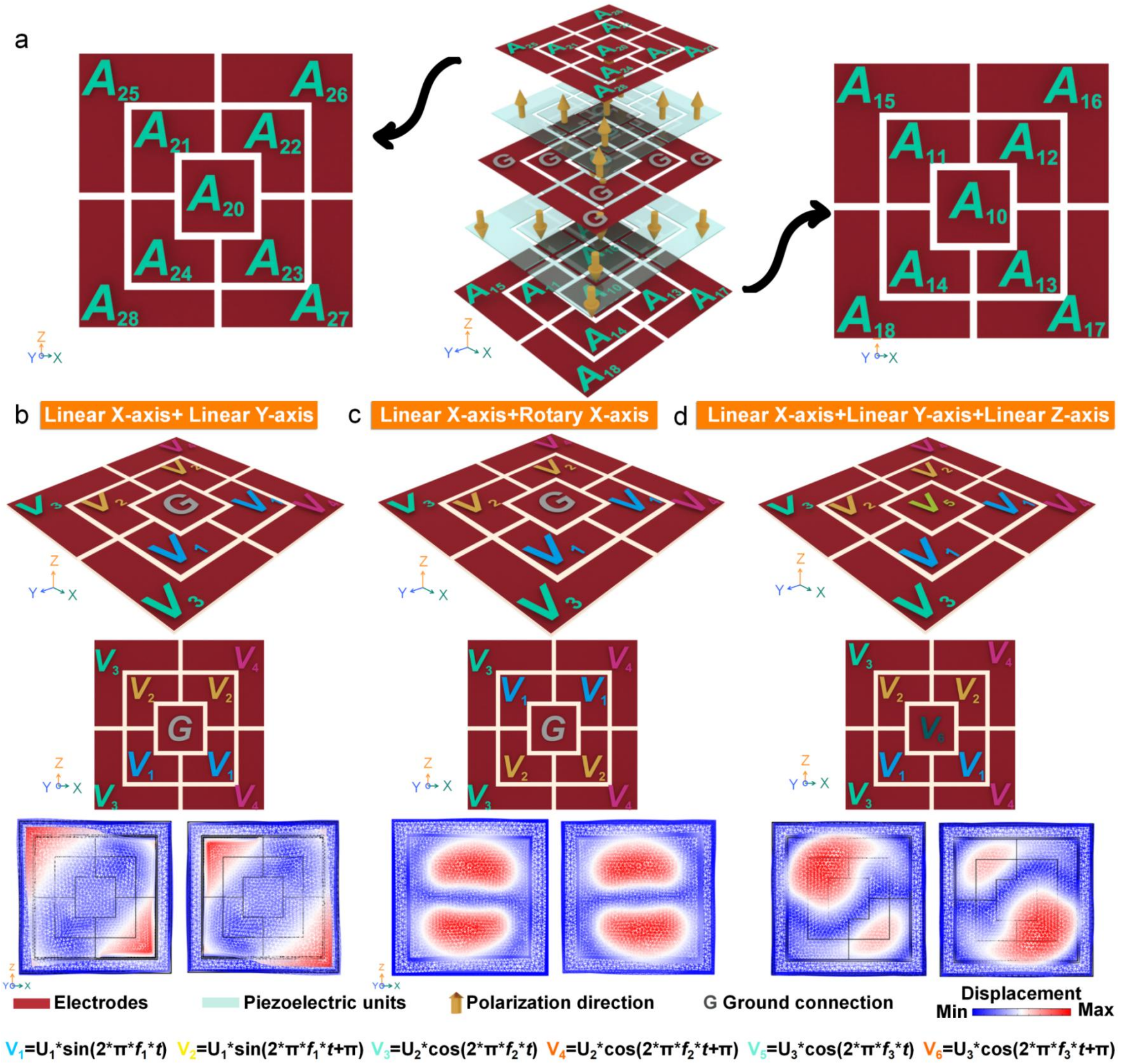


**Supplementary Fig. 3. Design of an (9 × 2) arrayed PM.** (a) Exploded figure of an (9 × 2) arrayed PM. (b-d) The applying voltage signals and simulated deformations of simultaneously accomplish (b) the linear motion along the X-axis and the linear motion along the Y-axis, (c) the linear motion along the X-axis and the rotary motion along the X-axis, (d) the linear motion along the X-axis, the linear motion along the Y-axis and the linear motion along the Z-axis.

(2) The method for simultaneously accomplishing three motion modes

As shown in **Supplementary Fig. 3**, the piezoelectric units of an 9 × 2 arrayed PM are divided into three groups, where $A_{10}$ and $A_{20}$ are classified as group I, the $A_{11}$, $A_{12}$, $A_{13}$, $A_{14}$, $A_{21}$, $A_{22}$, $A_{23}$ and $A_{24}$

are classified as group II, and the $A_{15}$, $A_{16}$, $A_{17}$, $A_{18}$, $A_{25}$, $A_{26}$, $A_{27}$ and $A_{28}$ are classified as group III. By applying predetermined electric fields, group I realizes the linear motion along the Z-axis, group II and group III can realize the linear motions along the X-, Y- and Z-axis, and/or the rotary motions around the X-, Y- and Z-axis. The three motion modes can be independently excited, so one or several motion modes can be simultaneously accomplished by freely matching. The applied voltage signals and simulated deformations of simultaneously accomplish three multiple motion modes is shown in **Supplementary Fig. 3**.

For example, for simultaneously accomplishing the linear motion along the X-axis, the linear motion along the Y-axis and the linear motion along the Z-axis (**Supplementary Fig. 3d**), the $A_{20}$ with voltage signal $V_5$ and the $A_{10}$ with voltage signal $V_6$ are classified as group I, the $A_{13}$, $A_{14}$, $A_{23}$ and $A_{24}$ units with voltage signal $V_1$ and the $A_{11}$, $A_{12}$, $A_{21}$ and $A_{22}$ units with voltage signal $V_2$ are classified as group II, and the $A_{15}$, $A_{18}$, $A_{25}$ and $A_{28}$ units with voltage signal $V_3$ and the $A_{16}$, $A_{17}$, $A_{26}$ and $A_{27}$ units with voltage signal $V_4$ are classified as group III. In this case, various motion modes can be produced for group I, II and III, i.e., group I generates the arching (or incurvaing) deformation along the positive (or negative) direction of the Z-axis, group II generates the lateral translation deformation along the Y-axis, group III causes the whole PM by the lateral translation deformation along the X-axis. Therefore, simultaneously accomplishing the linear motion along the X-axis, the linear motion along the Y-axis and the linear motion along the Z-axis is realized.

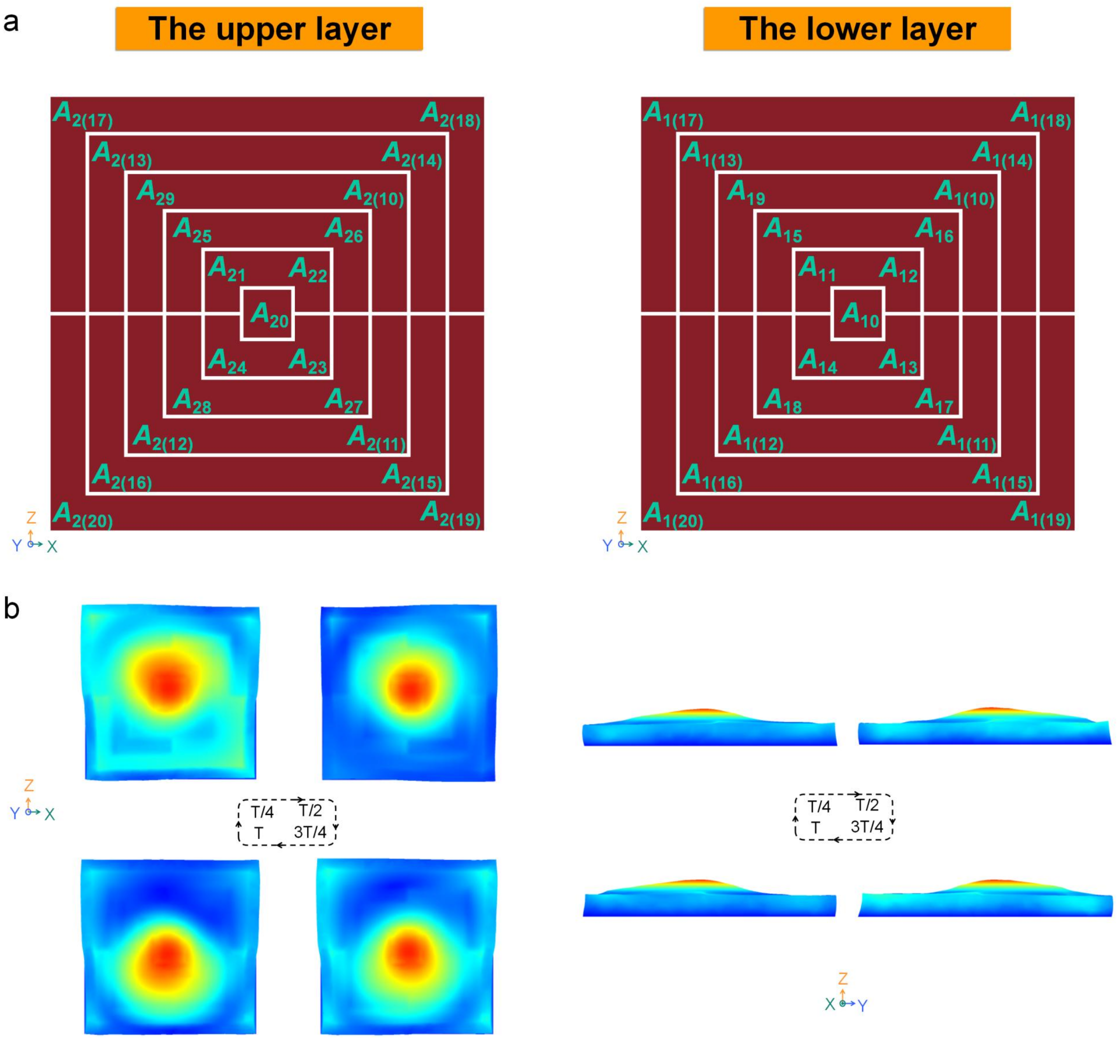


**Supplementary Fig. 4. Design of an (21 × 2) arrayed PM.** (a) Exploded figure of an (21 × 2) arrayed PM. (b) The deformation schematics of simultaneously accomplish six motion modes.

(3) The method for simultaneously accomplishing six motion modes

As shown in **Supplementary Fig. 4**, the piezoelectric units are divided into six groups, where $A_{10}$ and $A_{20}$ are classified as group I, the $A_{11}$, $A_{12}$, $A_{13}$, $A_{14}$, $A_{21}$, $A_{22}$, $A_{23}$ and $A_{24}$ are classified as group II, the $A_{15}$, $A_{16}$, $A_{17}$, $A_{18}$, $A_{25}$, $A_{26}$, $A_{27}$ and $A_{28}$ are classified as group III, the $A_{19}$, $A_{1(10)}$, $A_{1(11)}$, $A_{1(12)}$, $A_{29}$, $A_{2(10)}$, $A_{2(11)}$ and $A_{2(12)}$ are classified as group IV, the $A_{1(13)}$, $A_{1(14)}$, $A_{1(15)}$, $A_{1(16)}$, $A_{2(13)}$, $A_{2(14)}$, $A_{2(15)}$ and $A_{2(16)}$ are classified as group V, and the $A_{1(17)}$, $A_{1(18)}$, $A_{1(19)}$, $A_{1(20)}$, $A_{2(17)}$, $A_{2(18)}$, $A_{2(19)}$ and $A_{2(20)}$ are

classified as group VI. By applying predetermined electric fields, group I realizes the linear motion along the Z-axis, group II-V can realize the linear motions along the X-, Y- and Z-axis, and/or the rotary motions around the X-, Y- and Z-axis. The six motion modes can be independently excited, so one or several motion modes can be simultaneously accomplished by freely matching.

As shown in **Supplementary Fig. 4**, six motion modes can be simultaneously realized by more ordered units with the center outward expansion method.

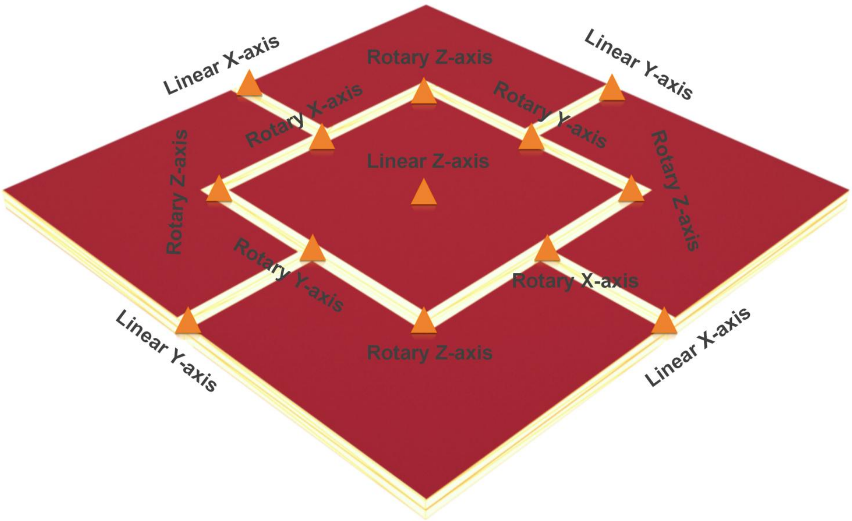


**Supplementary Fig. 5. Schematic diagram of test position of the desired motion modes.**

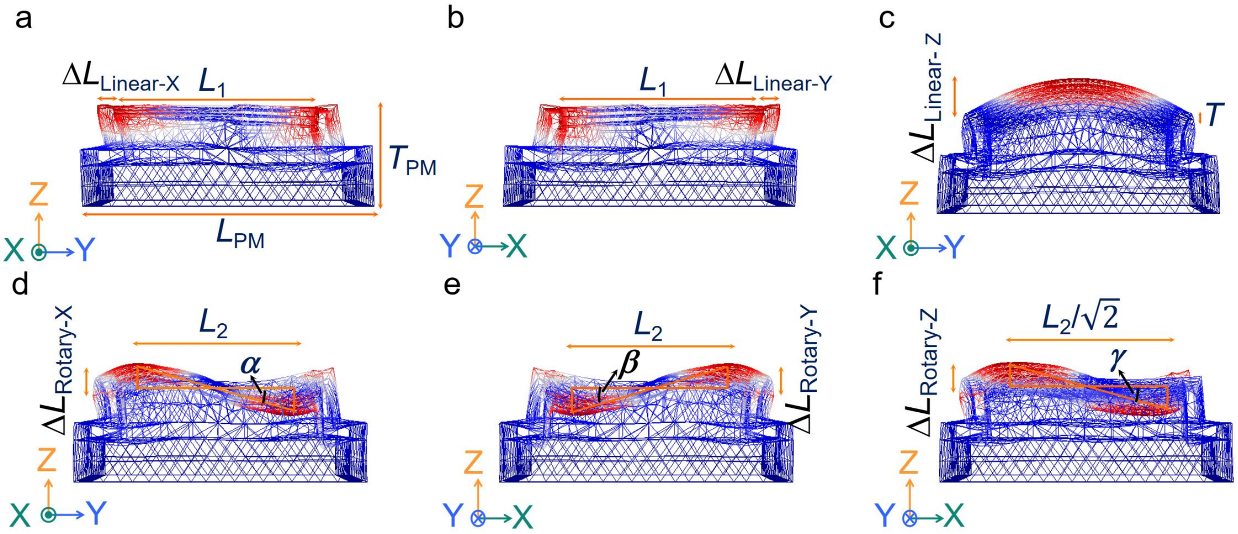


**Supplementary Fig. 6. The output performance of desired motion modes for PM by FEM simulation.** The simulated deformations of (a) the linear motion along the X-axis, (b) the linear motion along the Y-axis, (c) the linear motion along the Z-axis, (d) the rotary motion around the X-axis, (e) the rotary motion around the Y-axis, (f) the rotary motion around the Z-axis.

As shown in **Supplementary Fig. 6**, the output performance including the piezoelectric strains ($\varepsilon_1$, $\varepsilon_2$ and $\varepsilon_3$) and the rotation angles ($\alpha$, $\beta$, and $\gamma$) can be realized by exciting desired motion modes (including the linear motions along the X-, Y- and Z-axis and the rotary motions around the X-, Y- and Z-axis). Theoretically, the piezoelectric strains ($\varepsilon_1$, $\varepsilon_2$ and $\varepsilon_3$) of PM generating the linear motions along the X-, Y- and Z-axis can be calculated using the following formulas,

$$\varepsilon_1 = \frac{\Delta L_{\text{linear X}}}{L_{\text{PM}}} \tag{S1}$$

$$\varepsilon_2 = \frac{\Delta L_{\text{linear Y}}}{L_{\text{PM}}} \tag{S2}$$

$$\varepsilon_3 = \frac{\Delta L_{\text{linear Z}}}{T_{\text{PM}}} \tag{S3}$$

where $\Delta L_{\text{Linear-X}}$, $\Delta L_{\text{Linear-Y}}$ and $\Delta L_{\text{Linear-Z}}$ are the apparent displacement amplitude corresponding to different motion modes; $L_{\text{PM}}$ and $T_{\text{PM}}$ denote the width and height of PM, respectively. And the rotation angles ($\alpha$, $\beta$, and $\gamma$) of PM generating the rotary motions around the X-, Y- and Z-axis can be estimated by the following formulas,

$$\alpha = \tan^{-1}\frac{L_2/2}{L_{\text{Rotary X}}} \tag{S4}$$

$$\beta = \tan^{-1}\frac{L_2/2}{L_{\text{Rotary Y}}} \tag{S5}$$

$$\gamma = \tan^{-1}\frac{L_2/\sqrt{2}}{\Delta L_{\text{Rotary Z}}} \tag{S6}$$

where $\Delta L_{\text{Rotary-X}}$, $\Delta L_{\text{Rotary-Y}}$, and $\Delta L_{\text{Rotary-Z}}$ are the apparent displacement amplitude corresponding to different motion modes; $L_2$ denote the width of the $A_{331}$ or $A_{332}$ units (shown in **Supplementary Fig. 6**).

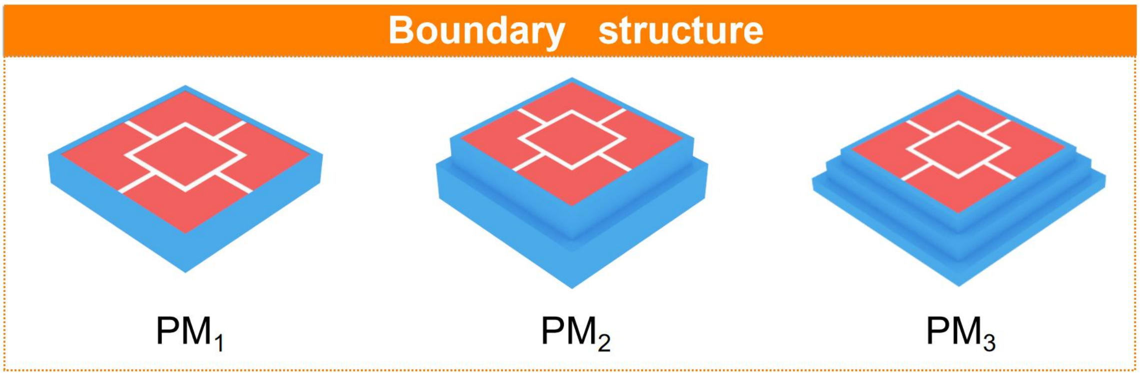


**Supplementary Fig. 7. Structure of the PM with the various boundary structures.**

**Supplementary Table 1. Comparison of the surface average stress of PM with the various boundary structures by FEM simulation.**

| Structures | The surface average stress (N/m$^2$) | |
|---|---|---|
| | Soft PZT ceramics | [001]-PIMNT single crystals |
| $PM_1$ | $8.13*10^5$ | $1.98*10^6$ |
| $PM_2$ | $7.64*10^6$ | $1.88*10^7$ |
| $PM_3$ | $1.74*10^6$ | $4.28*10^6$ |

## Supplementary Note 2. The output performance the PM-based ALENS.

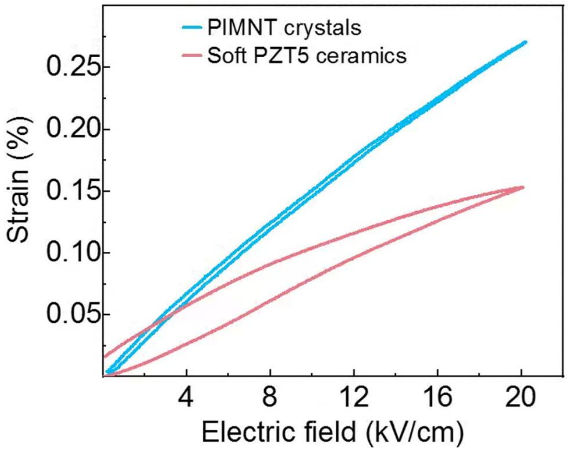


**Supplementary Fig. 8. The unipolar strain curves for the soft PZT5 ceramics and the PIMNT single crystals under 20 kV/cm at 1 Hz.**

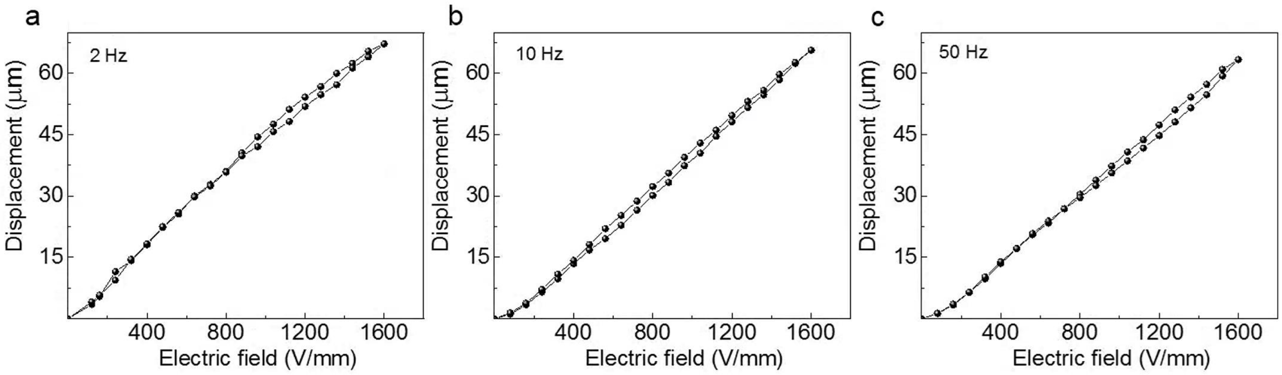


**Supplementary Fig. 9. The hysteresis characteristic of the PM-based ALENS under the different frequencies, including (a) 2 Hz, (b) 10 Hz, (c) 50 Hz.**

(1) The hysteresis of the piezoelectric materials

As shown in **Supplementary Fig. 8**, compared with the commercial PZT5 ceramics (~20%), the PIMNT single crystals have the advantage of very low strain hysteresis (~3%), reducing complexity and cost to the feedback control of piezoelectric devices. Meanwhile, the strain of the PIMNT single crystal is 0.27% at an electric field of 20 kV/cm, which is double the value of the soft PZT5

ceramics (~0.15%). Thus, in this work, we selected PIMNT as the piezoelectric elements to achieve high output strains.

(2) The hysteresis of the designed PM-based ALENS

The hysteresis in the ALENS is the main factor that affects its displacement accuracy, so the hysteresis curve of the PM-based ALENS was analyzed and measured (**Supplementary Fig. 9**). The calculated results show that the maximum hysteresis of the PM-based ALENS at 2 Hz, 10 Hz and 50 Hz are relatively low, being 3.9%, 3.8% and 4.2% respectively, which indicates that the PM-based ALENS exhibits high control accuracy and operation reliability.

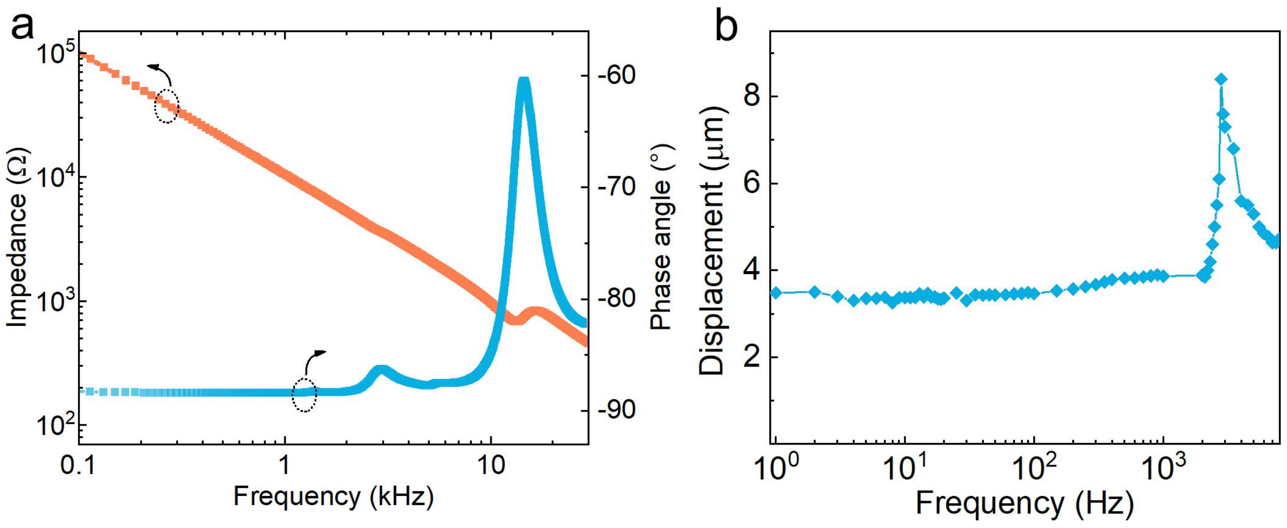


**Supplementary Fig. 10. The frequency characteristic.** (a) Impedance and phase spectra of the PM-based ALENS as a function of frequency ranging from 20 Hz to 30 kHz at room temperature. (b) Vibration displacement amplitudes of the PM-based ALENS for the AF under different frequencies.

(1) The response speed of the PM-based ALNES

The response speed is the degree of rapidity with which the ALENS transitions from one state to another after a given operation[1,2], which can be represented by the response time. It is calculated as follows[3],

$$t_{\text{response time}} = \frac{1}{f_{\text{operation frequency}}} \qquad \text{(S7)}$$

According to the above formula, the response time is closely related to the frequency of the operating electric field. As shown in **Supplementary Fig. 10a**, the impedance spectra have two obvious resonance peaks at 2.9 kHz and 13.8 kHz. In our present work, the PM-based ALNES works under the quasi-static condition, so its operating frequency is much lower than the resonance frequency. In order to acquire the minimum response time, we tested and added the output displacement of the PM-based ALENS under different frequencies into the experiment part. As

shown in **Supplementary Fig. 10b,** when the working frequency is below 2 kHz, the output displacement of the PM-based ALENS for the AF remains basically stable. Thus, the minimum response time of the PM-based ALNES is around 0.5 ms according to the **Equation (S7)**, which fulfills the requirement of optical systems.

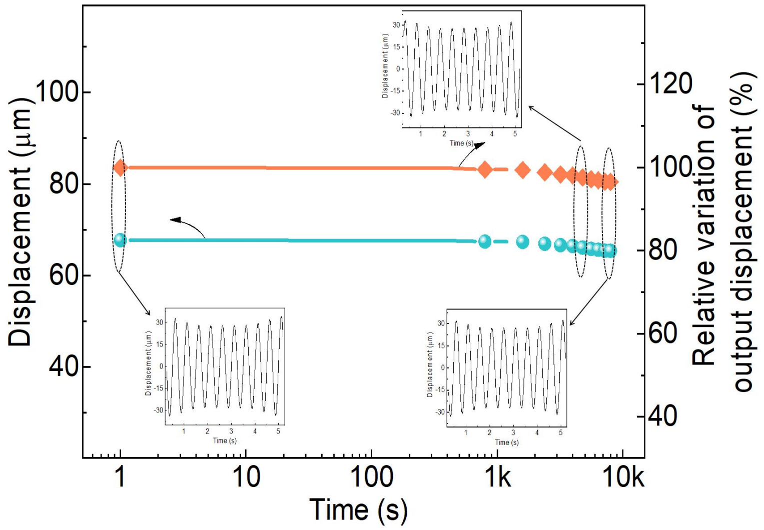


**Supplementary Fig. 11. Fatigue characteristics of the PM-based ALENS (the inset showing the transient surface displacement of the PM-based ALENS.**

Considering the stability and robustness, the fatigue characteristics of the PM-based ALENS were measured at 2 Hz under 1600 V/mm, as shown in **Supplementary Fig. 11**. The inset of (b) shows transient response of the displacement for the PM-based ALENS, where the output displacements exhibit good repeatability, rapid response, high controllability, and excellent stability. The output displacement of the PM-based ALENS maintains almost unchanged over 14400 vibration cycles in the durability test, representing its long-term robustness, high reliability and good stability.

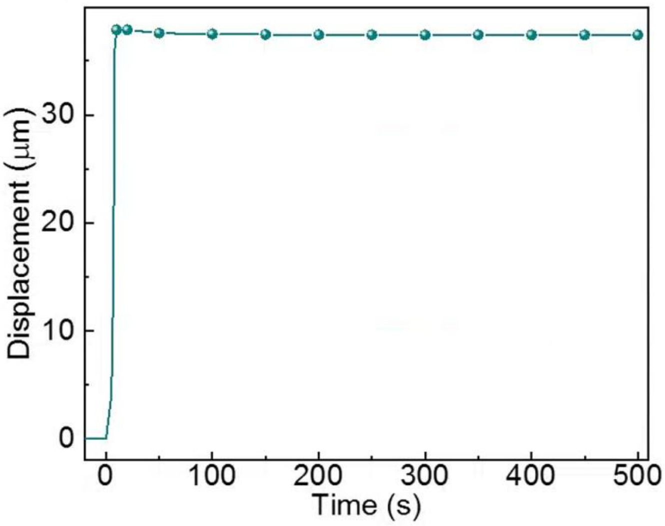


**Supplementary Fig. 12. Displacement of the PM-based ALENS when driven with a sudden voltage change (under 1000 V/mm).**

## Supplementary Note 3. Spot motion variation for the PM-based ALENS.

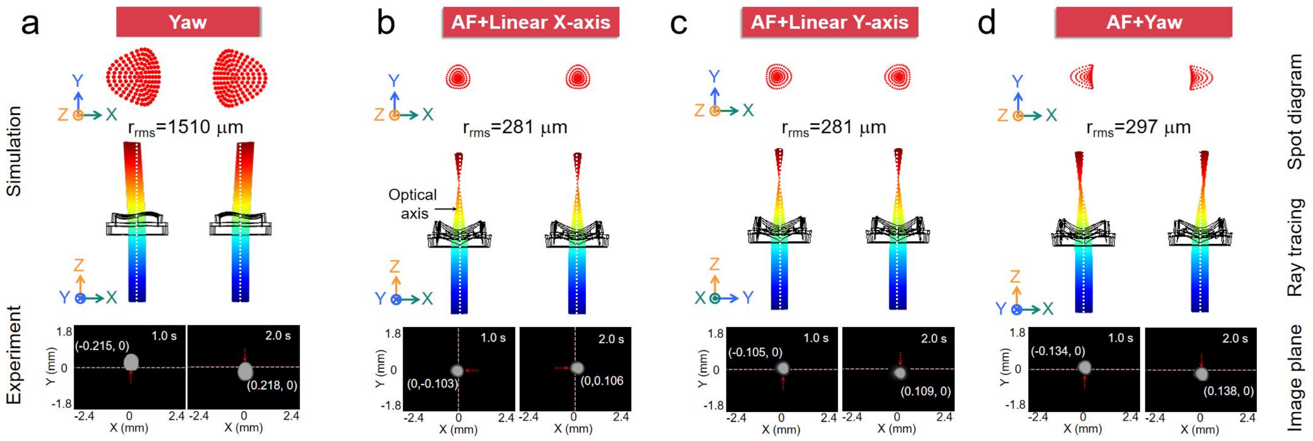


**Supplementary Fig. 13. The PM-based ALENS based on the AF and OIS functions under basic modes for spot motion variation.** Ray optics simulations (including ray tracing and spot diagram) and dynamic characteristics experiment of optical spot for (a) yaw, (b) the coupled modes of the AF and the linear motion along the X-axis, (c) the coupled modes of the AF and the linear motion along the Y-axis, (d) the coupled modes of the AF and yaw in the PM-based ALENS.

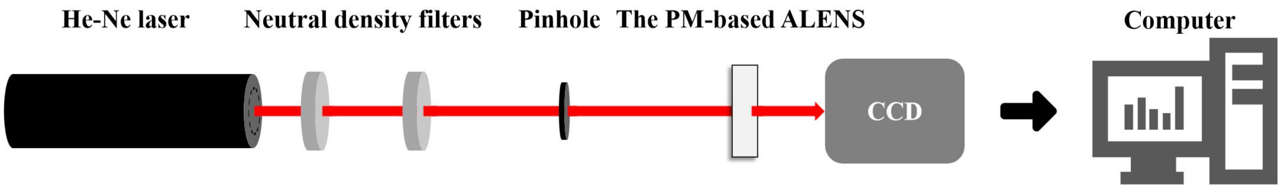


**Supplementary Fig. 14. The experimental setup for obtaining the spot size variations of the PM-based ALENS.**

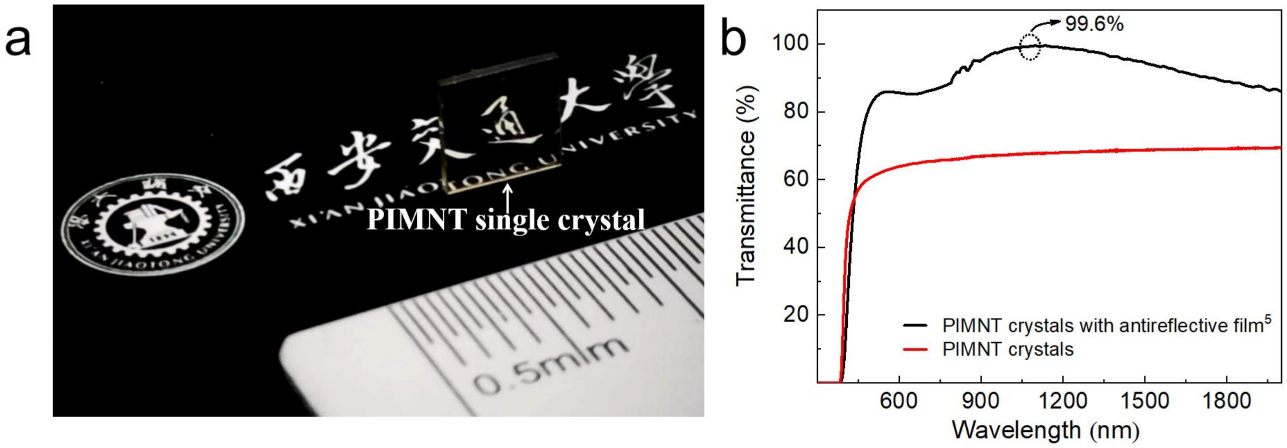


**Supplementary Fig. 15. (a) Photograph of transparent PIMNT crystal. (b) Light transmittance of transparent PIMNT crystals with and without antireflection film[5].**

As shown in **Supplementary Fig. 15a**, the PIMNT single crystal is clearly transparent. The light transmittance of the PIMNT single crystal is found to be very close to its theoretical limit (i.e., optical loss is only from surface reflection). The optical transmittance of transparent PIMNT crystals is about 70% in the wavelength range of 550 to 2000 nm (**Supplementary Fig. 15b**), which is close to lithium niobate single crystal ($LiNbO_3$, LN)[4]. In addition, we can greatly improve the crystal transmittance through the coating of antireflective film on the crystal surfaces. In particular, at a wavelength of 1064 nm the optical transmittance of PIMNT crystal can be enhanced up to 99.6%[5].

**Supplementary Table 2. The simulated and experimental values of the rotation angles $\alpha$, $\beta$ and $\gamma$, for the PM based on soft PZT ceramics and [001]-PIMNT single crystals under the electric field of 800 V/mm.**

| Motion modes | Simulated rotation angles (′) | | Experimental rotation angles (′) | |
|---|---|---|---|---|
| | PZT-5 | [001]-PIMNT | PZT-5 | [001]-PIMNT |
| $\alpha$-mode | 9.118 | 19.738 | 10.879 | 23.674 |
| $\beta$-mode | 9.118 | 19.738 | 11.315 | 20.743 |
| $\gamma$-mode | 2.742 | 6.886 | 2.796 | 6.836 |

**Supplementary References**


1. Abe, K. et al. The Electrostrictive unimorph for displacement control. *Jpn. J. Appl. Phys*. **21**. L408 (1982).
2. Zheng, L. et al. An ultra-sensitive and rapid response speed graphene pressure sensors for electronic skin and health monitoring. *Nano Energy*. **23**, 7-14 (2016).
3. Sun, C. L. et al. A novel drum piezoelectric-actuator. *Appl Phys A-Mater*. **84**, 385-389 (2006).
4. Dhar, A. et al. Optical properties of reduced lithium niobate single crystals. *J Appl. Phys*. **68**.5804-5809 (1990).
5. Liu, X. et al. Ferroelectric crystals with giant electro-optic property enabling ultracompact Q-switches. *Science* **376**. 371-377 (2022).
