# Supplementary material for: Designing transparent piezoelectric metasurfaces for adaptive optics
Source: Nat Commun. 2024 Jan 27;15:805. doi: 10.1038/s41467-024-45088-3 (PMC10821918; doi:10.1038/s41467-024-45088-3)
Supplement: Supplementary file 3 — Description of Additional Supplementary Files [file 41467_2024_45088_MOESM3_ESM.pdf]

DESCRIPTION FOR ADDITIONAL SUPPLEMENTARY FILES DOCUMENT

Supplementary Movie 1:

The dynamic deformation graphics of all the desired motion modes for the PM obtained by FEM simulation.

Supplementary Movie 2:

The PM-based ALENS based on the AF and OIS functions under the desired motion modes for spot motion variation.
