# Supplementary material for: Designing transparent piezoelectric metasurfaces for adaptive optics
Source: Nat Commun. 2024 Jan 27;15:805. doi: 10.1038/s41467-024-45088-3 (PMC10821918; doi:10.1038/s41467-024-45088-3)
Supplement: Supplementary file 6 — Reporting Summary [file 41467_2024_45088_MOESM6_ESM.pdf]

nature research

Corresponding author(s): Xiangyu Gao, Zhuo Xu, Fei Li

# Lasing Reporting Summary

Nature Research wishes to improve the reproducibility of the work that we publish. This form is intended for publication with all accepted papers reporting claims of lasing and provides structure for consistency and transparency in reporting. Some list items might not apply to an individual manuscript, but all fields must be completed for clarity.

For further information on Nature Research policies, including our data availability policy, see Authors & Referees.

## Experimental design

**Please check: are the following details reported in the manuscript?**

1. Threshold

Plots of device output power versus pump power over a wide range of values indicating a clear threshold

☐ Yes
☒ No

In the manuscript, the test results dose not related to the lasing.

2. Linewidth narrowing

Plots of spectral power density for the emission at pump powers below, around, and above the lasing threshold, indicating a clear linewidth narrowing at threshold

☐ Yes
☒ No

In the manuscript, the test results dose not related to the lasing.

Resolution of the spectrometer used to make spectral measurements

☐ Yes
☒ No

In the manuscript, the test results dose not related to the lasing.

3. Coherent emission

Measurements of the coherence and/or polarization of the emission

☐ Yes
☒ No

In the manuscript, the test results dose not related to the lasing.

4. Beam spatial profile

Image and/or measurement of the spatial shape and profile of the emission, showing a well-defined beam above threshold

☐ Yes
☒ No

In the manuscript, the test results dose not related to the lasing.

5. Operating conditions

Description of the laser and pumping conditions
*Continuous-wave, pulsed, temperature of operation*

☐ Yes
☒ No

In the manuscript, the test results dose not related to the lasing.

Threshold values provided as density values (e.g. W $cm^{-2}$ or J $cm^{-2}$) taking into account the area of the device

☐ Yes
☒ No

In the manuscript, the test results dose not related to the lasing.

6. Alternative explanations

Reasoning as to why alternative explanations have been ruled out as responsible for the emission characteristics
*e.g. amplified spontaneous, directional scattering; modification of fluorescence spectrum by the cavity*

☐ Yes
☒ No

In the manuscript, the test results dose not related to the lasing.

7. Theoretical analysis

Theoretical analysis that ensures that the experimental values measured are realistic and reasonable
*e.g. laser threshold, linewidth, cavity gain-loss, efficiency*

☐ Yes
☒ No

In the manuscript, the test results dose not related to the lasing.

8. Statistics

Number of devices fabricated and tested

☐ Yes
☒ No

In the manuscript, the test results dose not related to the lasing.

Statistical analysis of the device performance and lifetime (time to failure)

☐ Yes
☒ No

In the manuscript, the test results dose not related to the lasing.



March 2018
